# Supplementary material for: Collaborative intelligence in AI: Evaluating the performance of a council of AIs on the USMLE
Source: PLOS Digit Health. 2025 Oct 9;4(10):e0000787. doi: 10.1371/journal.pdig.0000787 (PMC12510544; doi:10.1371/journal.pdig.0000787)
Supplement: S2 File — (ZIP) [file pdig.0000787.s002.zip › S2 Data Raw data files containing unprocessed question inputs and LLM outputs including transcripts of deliberations/STEP3_Question-97-1_Type-MC-NJ.docx]

**Facilitator** **[Round 1]:** A 29-year-old woman, gravida 3, para 3, comes to the office because of a 1-year history of recurrent low back pain. Rest and analgesics typically resolve the pain within 2 weeks. However, the pain recurs every 2 to 3 months. Medical history is remarkable for gestational diabetes during her pregnancies. She takes no medications except for an oral contraceptive. She walks 3 miles daily for exercise and works as a sales representative for a computer software company. She is 165 cm (5 ft 5 in) tall and weighs 100 kg (220 lb); BMI is 37 kg/m². Vital signs are normal, and physical examination discloses no abnormalities. Which of the following is the most appropriate next step? (A) Administer an epidural injection of methylprednisolone (B) Order MRI of the lumbosacral spine (C) Order x-rays of the lumbosacral spine (D) Recommend beginning a weight loss program (E) Recommend decreasing physical activity

**AI_C:** (1) RESTATING THE QUESTION: A woman in her late twenties, who has had three successful pregnancies, has been experiencing recurrent lower back pain over the past year. She has been able to alleviate her discomfort with rest and over-the-counter pain medications, but the problem continues to recur every few months. This woman also has had gestational diabetes during her pregnancies. The patient is using only an oral contraceptive pill for treatment, and exercises regularly by walking. With a height of 165 cm and weight of 100 kg, her computed BMI is classified as obesity. Her vitals and a physical exam showed no abnormalities. The question asks for the best next step in managing her condition among options of different treatments and diagnostic tests. (2) PLANNING: The presented information makes us consider two possible explanations for the persistent back pain she is suffering from: mechanical, possibly caused by obesity and physical stress, or pathological, such as musculoskeletal or neurological disorders. Analgesics and rest briefly relieve the pain, pointing to a mechanical rather than pathological cause. Taking into account her BMI of 37 kg/m², we could consider the idea that the recurring back pain may be a result of her weight. (3) CLINICAL REASONING: It's common to see patients who are obese suffering from lower back pain due to the increased load and stress on the spine. Obesity alters the normal curvature and alignment of the spine, leading to an increased chance of spinal disorders such as disc herniation or spinal stenosis. It's also important to note that the woman doesn't show any signs of alarms like fever, night sweats, recent trauma, bowel or bladder incontinence, or neurological deficits, situations that might have indicated a more serious condition, and would have justified using diagnostic tests or other therapeutic alternatives such as MRI, epidural injections, or x-rays. (4) CONCLUDING: The woman is obese and has recurrent pain in the lower back, which seems to be mechanical in nature, rather than pathological. Before recommending any invasive diagnostics or treatments, it's important to address the probable root cause of her pain: obesity. (5) RESPONSE: Therefore, the most clinical logical response to this scenario is (D) Recommend beginning a weight loss program. This would not only potentially relieve her recurring back pain but also help her on a general health level considering her history of gestational diabetes.

**AI_A:** 1) RESTATING THE QUESTION: This question presents a 29-year-old overweight woman who has had recurrent low back pain for a year. The pain comes and goes, resolving with rest and analgesics but returns every few months. She has a history of gestational diabetes and takes oral contraceptives. She is physically active, walking 3 miles daily and works in a non-physical job. Despite having a body mass index (BMI) of 37 kg/m² which indicates obesity, her vital signs are normal and there are no abnormalities noted on physical examination. The question asks for the most appropriate next step in her management. 2) PLANNING: The woman's complaints of recurrent low back pain and her medical history of gestational diabetes, current overweight state, high BMI indicating obesity, and her intake of oral contraceptives might suggest a number of potentially associated conditions. It may be assumed that her pain is due to a structural or neurological issue in her spine since she is having low back pain which might lead to options like MRI or x-ray of the spine or epidural injection of steroid. Alternatively, one could assume the pain is due to increased mechanical load from excess weight because of her obesity. This would suggest a weight reduction program. 3) CLINICAL REASONING: Considering her age and the recurrent nature of her symptoms, a structural or persistent neurological pathophysiology seems less likely. Although overweight and obesity can be associated with an increased risk of back pain due to the added strain on the back muscles and ligaments, it should also be noted that obesity can increase the risk of type 2 diabetes along with her history of gestational diabetes. Further rest and analgesics are resolving her back pain which also suggests it's less likely due to a severe and grave structural or neurological issue. 4) CONCLUDING: Given the provided information about her physical strain due to obesity and recalling the connection between obesity, type 2 diabetes, and her history of gestational diabetes, it seems more likely that her recurrent low back pain is due to mechanical load rather than a severe spinal or neurological condition. Considering the facts that she is physically active and she has a somewhat sedentary job, which are not supportive of drastic spinal issues, further radiological diagnosis or aggressive pharmacological interventions are not needed here. 5) RESPONSE: It seems that the most appropriate next step would be to (D) Recommend beginning a weight loss program, as it would help reduce the mechanical load on her back, cause a decrease in her back pain, and also provide additional health benefits considering her high risk for developing type 2 diabetes.

**AI_B:** RESTATING THE QUESTION: The description given portrays a young woman experiencing recurrent episodes of lower back pain. Regardless of her healthy physical activity and seemingly normal vital signs, she is overweight with a Body Mass Index (BMI) greater than 35 kg/m2, placing her in the obese category. Her medical history is noteworthy for gestational diabetes, which she might have experienced due to her increased body mass. Now, the task is to identify the most suitable next step in her clinical management, from options including invasive procedures, diagnostic imaging, recommendation of a weight loss program or reduction in physical activity. PLANNING: Back pain is commonly related to musculoskeletal issues often associated with poor postural habits, occupational biomechanics, and obesity. Given the ability of rest and analgesics to resolve this patient's pain, it is plausible that her symptoms are due to such musculoskeletal misalignments exacerbated by her weight status. Assumption here is that weight loss could alleviate part of the strain on her back, leading to a decreased frequency of recurrent pain. CLINICAL REASONING: Radiological imaging or an epidural injection should be reserved for patients with a history indicative of severe neurological issues, inflammatory back pain or malignancies, none of which this patient exhibits. Furthermore, because she maintains a relatively active lifestyle, suggesting to decrease her physical activity seems counter-productive for an obese person. Indeed, obesity can put an enormous amount of stress on the back and other weight-bearing parts of the body. This stress, especially on the disks and muscles of the lower back, can cause chronic pain. In this context, weight reduction can relieve some of this stress. CONCLUDING: In light of the details provided by the patient's history and physical examination, both her documented obesity and her lack of alarming symptoms suggest that a conservative approach targeting weight loss would likely be effective in reducing the frequency and intensity of her back pain episodes. RESPONSE: Based on the clinical findings and her lifestyle, option (D) "Recommend beginning a weight loss program" would be the most appropriate next step in this case. Introducing a weight management plan could help mitigate the back pain by reducing the stress carried by her lumbosacral spine without the need for invasive procedures or reducing her level of physical activity.

**AI_E:** RESTATING THE QUESTION: This case presents a 29-year-old woman who is gravida 3, para 3, suffering from a 1-year history of recurrent low back pain. The pain gets resolved with rest and analgesics typically in two weeks, and recurs every 2 to 3 months. Her medical history includes gestational diabetes during her pregnancies. She is on oral contraceptives and maintains a regular exercise routine that involves a daily walk of three miles. Professionally, she is a sales representative at a computer software company. She has a BMI of 37 kg/m², which classifies her as obese. She has no apparent abnormality in her physical examination, and her vital signs are normal. The question seeks the most appropriate next step to take benefiting her health condition, with options including administration of an epidural injection of methylprednisolone, ordering an MRI or x-rays of the lumbosacral spine, recommending a weight loss program, or recommending reduced physical activity. PLANNING: The primary assumptions underline that the recurrent low back pain might be associated with her overweight status, associated posture issues, or any underlying spinal complications. However, the pain's temporary resolution with rest and analgesics suggests it could be more of a musculoskeletal or weight-related issue rather than a severe clinical condition needing immediate invasive procedures or imaging. CLINICAL REASONING: Given repetitive pain, it's reasonable to consider that a spinal disorder, which might require an MRI or x-rays, could be a potential reason. However, those tests are typically reserved for symptoms that suggest more severe conditions, like significant trauma, weight loss, history of cancer, fever, intravenous drug use, pain lasting more than six weeks, or pain at night or at rest. The patient does not exhibit these signs; therefore, these assumptions are less likely. The assumption that the pain is weight-related leads to the rationale that losing weight might reduce the burden on her back, potentially alleviating the pain. CONCLUDING: Thus, the best clinical approach would be to focus firstly on factors that can be modified without involving invasive or expensive procedures, given the absence of alarming signs in her case. RESPONSE: So, in the context of this patient's history and clinical findings, the most appropriate next step to manage her recurrent low back pain should be (D) Recommending a weight loss program.

**AI_D:** 1) RESTATING THE QUESTION: To paraphrase, a 29-year-old woman with a medical history of gestational diabetes has been suffering from recurrent low back pain for a year now. Being overweight, she takes no medications except for an oral contraceptive. Despite having normal vital signs and physical examination findings, the question asks what the most relevant next step in her care would be considering her symptom of recurrent back pain, which is managed by rest and analgesics within 2 weeks every time but, regrettably, recurs every 2 to 3 months. 2) PLANNING: The assumptive approach is: 1) Her back pain is occurring due to a musculoskeletal or nerve-related issue, warranting diagnostics (options B and C) or direct treatment (option A). 2) Her back pain is occurring within the context of her lifestyle, such as her weight (option D) or amount of physical activity (option E). Considering the given information, a musculoskeletal or nerve-related issue seems unlikely given the lack of abnormal findings in the physical examination. I suggest giving priority to the hypothesis that the recurrent pain is significant within her lifestyle context, considering her high BMI and her occupation as a sales representative which might involve prolonged sitting or possibly carrying items, both potentially causing strain on the lower back. 3) CLINICAL REASONING: This patient's BMI of 37 kg/m² indicates obesity, which can contribute to low back pain by increasing the load on the spinal column and the pressure on the intervertebral discs, potentially causing disc degeneration over time. Notably, the patient's pain subsides with rest and returns every 2-3 months. This pattern of pain supports the idea that external factors, such as physical activity, might play an important role. Her work setting as a sales representative could entail prolonged static postures or carrying heavy items, both leading to mechanical back pain due to strain and possible subsequent inflammation. While exercise such as her daily walks is generally beneficial, carrying excess weight can still give rise to biomechanical strain. On the other hand, her gestational diabetes and oral contraceptive usage are not typically associated with causing consistent low back pain over a long term. 4) CONCLUDING: In absence of any abnormal findings on physical examination, and considering the risk factors in her lifestyle such as obesity and possible prolonged sedentary periods or strain at work, it is reasonable to address her weight first and its potential role in her recurring back pain. Her recurring pain that temporary resolves on resting further suggest mechanical nature of the pain rather than a pathological problem with her lumbosacral spine, making the diagnostic investigations and direct symptomatic treatments less crucial in this stage. 5) RESPONSE: Therefore, the most appropriate next step is to recommend beginning a weight loss program (Option D). This could alleviate the strain on her lower back by reducing the loading intensity and hence mitigate her recurrent pain. It is crucial, however, to tailor this weight loss program to her individual needs, preferences, and existing exercise levels.

**Facilitator** The consensus among the AI responses is that the most appropriate next step in managing the 29-year-old woman's recurrent low back pain is to recommend beginning a weight loss program. This conclusion is based on her medical history, lifestyle, and current health status. The woman is obese, with a BMI of 37 kg/m², and has a history of gestational diabetes. She experiences recurrent low back pain, which resolves with rest and analgesics but returns every few months. Despite her obesity, her vital signs are normal, and there are no abnormalities noted on physical examination. The AI responses suggest that her back pain is likely due to the mechanical load from her excess weight rather than a severe spinal or neurological condition. This is supported by the fact that her pain subsides with rest and analgesics, indicating it's less likely due to a severe and grave structural or neurological issue. Furthermore, her physically active lifestyle and sedentary job do not support the idea of drastic spinal issues. Therefore, the AI responses agree that recommending a weight loss program would be the most beneficial next step. This would not only potentially alleviate her recurring back pain by reducing the mechanical load on her back but also provide additional health benefits considering her high risk for developing type 2 diabetes due to her history of gestational diabetes and current obesity. The AI responses also agree that invasive procedures, diagnostic imaging, or reduction in physical activity are not necessary at this stage.
